# Banana Peel Based Cellulose Material for Agriculture and Aquiculture: Toward Circular Economy

**DOI:** 10.3390/polym17091230

**Published:** 2025-04-30

**Authors:** Iris N. Serratos, Juan Antonio García Torres, Jorge Luis Mendoza Téllez, David Silva Roy, Ana María Soto Estrada, Norma Elena Leyva López, Hervey Rodríguez González, Sylvie Le Borgne, Karla Lorena Sánchez-Sánchez, Rebeca Sosa Fonseca

**Affiliations:** 1 Departamento de Química, Universidad Autónoma Metropolitana-Iztapalapa, Av. San Rafael Atlixco 186, Col. Vicentina, Ciudad de México 09340, Mexico; 2 Departamento de Física, Universidad Autónoma Metropolitana-Iztapalapa, Av. San Rafael Atlixco 186, Col. Vicentina, Ciudad de México 09340, Mexico; 3 Instituto Politécnico Nacional, Centro Interdisciplinario de Investigación para el Desarrollo Integral Regional, Unidad Sinaloa, Departamento de Biotecnología Agrícola, Guasave 81101, Mexico; 4 Departamento de Procesos y Tecnología, Universidad Autónoma Metropolitana-Cuajimalpa, Vasco de Quiroga 4871, Ciudad de México 05348, Mexico; 5 Departamento de Sistemas de Información y Comunicaciones, Universidad Autónoma Metropolitana-Lerma, Av. de las Garzas 10, Lerma de Villada 52005, Mexico

**Keywords:** compostable biopolymer, banana peels, agriculture, aquiculture, circular economy

## Abstract

This study explores the creation and characterization of a compostable biopolymer derived from banana peels, addressing the issue of organic waste. Rich in protein, fiber, water, and cellulose, banana peels can be transformed into biodegradable polymers through acid hydrolysis, which breaks down cellulose chains, making them suitable for use in aquiculture and agriculture. Methionine, an essential amino acid for aquiculture, was added to enhance the biopolymer’s value in fish feed. The biopolymer was synthesized by heating, crushing, and subjecting the peels to acid hydrolysis. The methionine was integrated by causing it to form ester bonds with the cellulose. The products were characterized using UV-VIS and IR spectroscopy, thermogravimetric analysis (TGA), and scanning electron microscopy (SEM). UV-VIS and IR spectra confirmed the incorporation of the methionine, while TGA showed reduced mass loss in the methionine-enriched biopolymer, likely due to the retention of water molecules. SEM images revealed roughness, indicating the crosslinking of the small cellulose chains. The incorporation of methionine led to a more uniform and compact structure. The obtained biopolymer has potential applications in agriculture, especially for potato cultivation, and shows promise for sustainable aquiculture, particularly in tilapia feed. This research contributes to both waste valorization and the development of eco-friendly materials.

## 1. Introduction

With the global population projected to increase to 9 billion by 2050, the amount of organic waste generated by human and agricultural activities is also expected to rise, creating environmental issues. The United Nations Millennium Development Goals emphasize the urgent need for action in ecological conservation, sustainable development, and poverty reduction. However, despite significant efforts by both national and international organizations, organic waste treatment alone will not be sufficient to address these challenges effectively [1]. In addition, bioeconomy strategies based on transforming biomass and organic waste into valuable products such as fuels, feed, renewable chemicals, biopolymers, and others have become an increasing reality in different regions of the world since the early 2000s [2].

**Figure 1:**
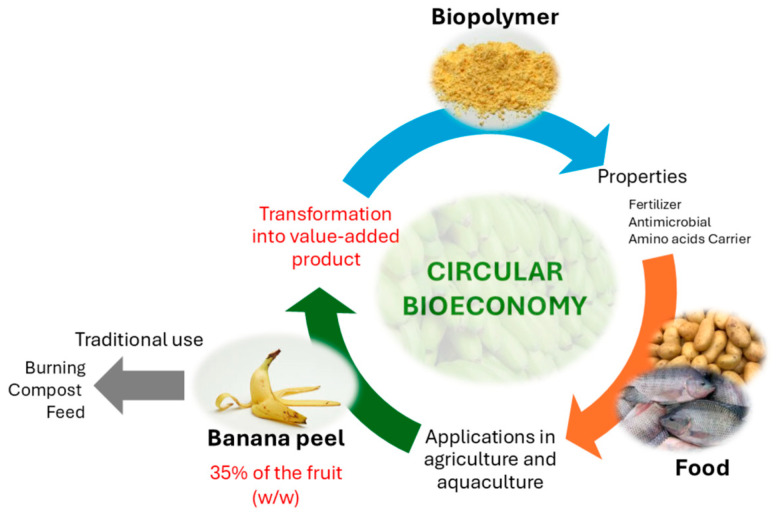
Circular bioeconomy.

Bananas are among the most widely cultivated crops in tropical countries. Fiallos-Cárdenas et al. (2022) [3] have depicted a circular bioeconomy system around the banana value chain, in which residual banana biomass could be used to produce enough energy to meet primary production requirements and bio-based products from banana leaves, pseudostems, rachis, and banana peel, which account for 35–50% of the banana weight [4], are commonly discarded as waste in municipal landfills. However, this problem can be mitigated by utilizing its valuable compounds as the dietary fiber fraction [5] or transforming the peels into higher-value-added by-products as is proposed in the present work (Figure 1).

Banana peel is primarily composed of biopolymers, including cellulose, hemicellulose, lignin, pectin, chlorophyll, fiber, and other low-molecular-weight substances [6,7,8]. Due to its high cellulose content (18–59%) [9], banana peel waste is a promising candidate for the development of biomaterials. Although the preparation of biomaterials has been explored similarly using other organic peels [3,10,11,12], in this study, we propose leveraging this waste to prepare biomaterials with potential applications in the agricultural and aquiculture sectors, providing an alternative use for this abundant waste.

Bishnoi et al. (2023) [7] reviewed studies demonstrating the creation of various value-added products using banana peel waste, taking advantage of the peels’ intrinsic properties [13]. Currently, there are direct applications for banana peels, such as their use in animal feed, such as for livestock in Uganda [14]. Similarly, banana peels are employed as a biofertilizer in compost, improving soil fertility and quality by providing essential nutrients, such as potassium and nitrogen [15]. Furthermore, these can be indirectly utilized by extracting valuable compounds [16] for energy production [17], antioxidants for medicinal purposes [18], and protein and fiber for the enzyme industry [19]. Recent studies have also explored the production of nanoparticles from hemicellulose, lignin, and pectin, which can serve as biomaterial stabilizers [6].

This approach aligns with the principles of a low-carbon circular bioeconomy, which include (1) optimizing substrate use, (2) minimizing greenhouse gas emissions, (3) reducing reliance on fossil fuels, (4) utilizing various industrial wastes, and (5) producing biodegradable products [20,21]. Considering the wide range of possibilities for reusing banana peel waste, our work primarily focuses on the fourth and fifth principles [16].

The goal of a circular bioeconomy is to promote the efficient use of resources, including waste and by-products, to sustainably produce value-added products, such as biofuels, biomaterials, biochemicals and food. The biomaterials derived from banana peel waste could provide valuable applications in both the agriculture and aquiculture industries [21,22,23]. The advances in the application of the biopolymer to date are as follows: the biopolymer has been applied to potato crops inoculated with the bacteria *Pectobacterium brasiliense*, reducing the incidence and severity of potato rot treated with this biomaterial. These results confirm that the biopolymer derived from banana peels has an antimicrobial effect. Regarding the application of this biopolymer to the growth of tilapia juveniles, it is being used as a binder in balanced feed. This application resulted in a significant improvement in the growth of tilapia juveniles over a 45-day period. Subsequently, the biopolymer containing methionine (an amino acid) will be tested in the nutrition of tilapia juveniles.

The use of banana peels as a biomaterial originated from a project by physicist Juan Antonio García Torres from Universidad Autónoma Metropolitana Unidad Iztapalapa. After preparing the biopolymer, its physical properties were analyzed and its potential as a plastic substitute was identified. The project aligns with the 2030 Agenda, highlighting Target 12.5 (waste reduction) and Target 12.2 (the efficient use of natural resources).

Using these types of materials to replace those that come from chemical processes, some of which can be highly polluting, is very important for maintaining biodiversity since biomaterials do not pollute nature when discarded after use. Reusing agricultural biomass helps reduce organic waste and promote the circular economy.

## 2. Materials and Methods

### 2.1. Reagents and Equipment

The following materials and equipment were used: banana peels, methionine (MilliporeSigma, Burlington, MA, USA), deionized water, hydrochloric acid (HCl), a FT-IR Spectrum GX, PerkinElmer Inc., Shelton, CT, USA, a UV-VIS GENESYS 180, Thermo Fisher Scientific Inc., Waltham, MA, USA, a Thermogravimetric analyzer (TGA) Diamond, PerkinElmer, Inc., Shelton, CT, USA, a spectrophotometer (Fluorescence), PerkinElmer Inc., 650-10S Shelton, CT, USA and a JEOL Ltd., JSM-7600 F, Akishima, Japan.

### 2.2. Synthesis of Biopolymer from Banana Peels

#### 2.2.1. Biopolymer Without Methionine

**Figure 2:**
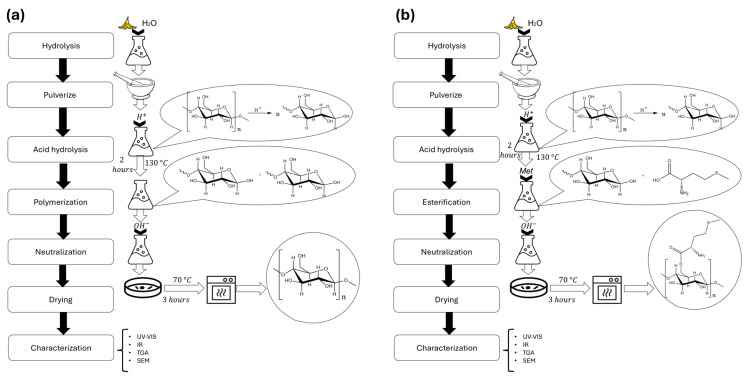
Comparison of the synthesis methods to obtain both biopolymers. (**a**) Diagram of process to obtain biopolymer without methionine. (**b**) Diagram of process to obtain biopolymer with methionine.

We first prepared the biopolymer by hydrolyzing the banana peels through heating. The peels were subsequently crushed into a fine paste to achieve a homogeneous consistency. Once the paste was uniform, acid hydrolysis was performed at pH = 1 using 0.5 M HCl and maintaining the temperature at 130 °C while stirring continuously for 2 h. Subsequently, the system was neutralized by adding 0.5 M NaOH until pH = 7, while continuously stirring. Then, we dried the biopolymer at 70 °C for 3 h (Figure 2a).

#### 2.2.2. Biopolymer with Methionine

Following the same steps to obtain the biopolymer, we added amino acid (6% relative to the pure biopolymer) to the system during the acid hydrolysis process. Under these conditions, esterification was expected to occur, leading to the binding of methionine to the primary alcohol group of glucose. The reaction conditions were maintained for an additional 2 h to ensure complete esterification.

Following the esterification step, the reaction was neutralized while stirring. The biopolymer was then dried at 70 °C for 3 h (Figure 2b).

### 2.3. Characterization of Biopolymer

The resulting product was characterized by using a UV-VIS GENESYS 180, Thermo Fisher Scientific Inc., Waltham, MA, USA from 330 nm to 800 nm; the samples were suspended in a proportion of 0.1 g of polymer over 2.5 mL of water to perform the measurements.

The FTIR characterization was performed with a FT-IR Spectrum GX, PerkinElmer Inc., Shelton, CT, USA. We performed the measurements using the ATR incorporated in the equipment, limiting between 4000 and 400 cm^−1^.

Thermal analysis of the polymer sample was performed considering a temperature range of 25 °C to 400 °C with an increase in temperature of 10 °C per minute, to mainly observe the stability of the biomaterial. We used a thermal analyzer for thermogravimetric (TG) and derivative thermogravimetric (DTG) analyses using a Diamond, PerkinElmer, Inc., Shelton, CT, USA.

Furthermore, we characterized the biopolymer by scanning electron microscopy (SEM) using the JEOL Ltd, JSM-7600 F, Akishima, Japan, with a power setting of 15 KV to obtain the images. Due to the samples not being conductive, before obtaining the SEM images, the samples were coated with gold by three exposures of 60 s using the Sputtering Denon Vacuum Co., Ltd., Japan. Additionally, with the same equipment, we obtained the energy-dispersive spectroscopy (EDS) to determine the composition of the biopolymer with and without methionine.

## 3. Results and Discussion

### 3.1. UV-VIS

**Figure 3:**
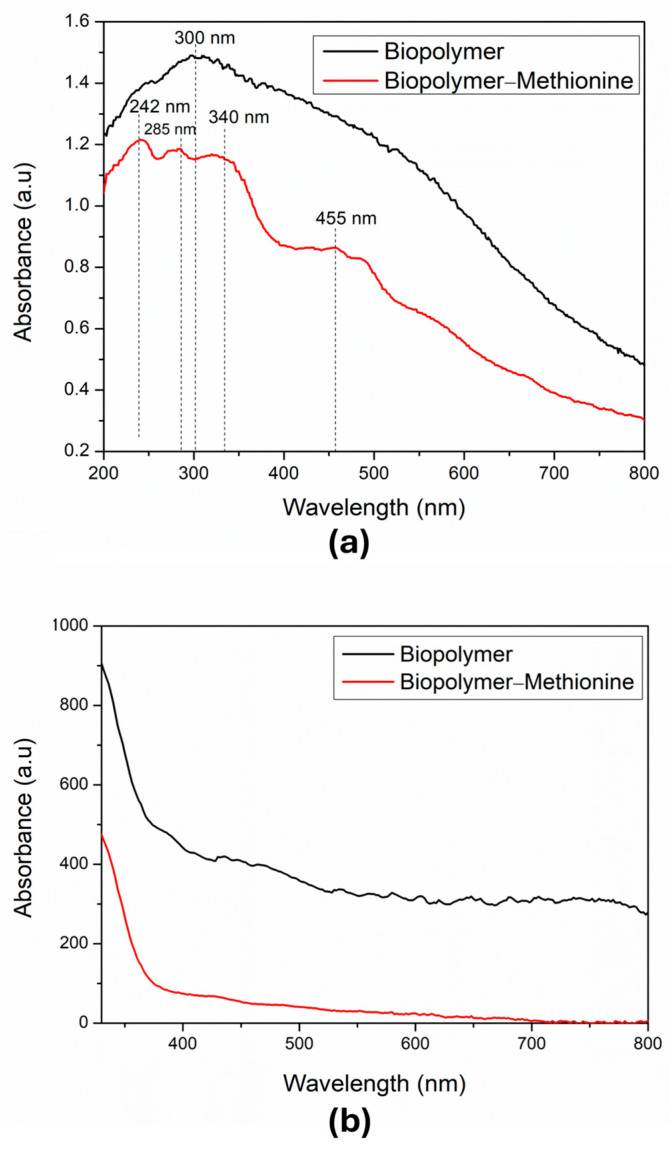
UV-VIS studies: (**a**) Absorption spectra of the biopolymer powder without (black line) and with methionine (red line). (**b**) Suspension of the natural banana biopolymer (black line) and suspension of the natural banana biopolymer with methionine (red line). These last two samples were suspended in water.

The UV-VIS spectra were measured by diffuse reflectance in the range from 200 nm to 800 nm. The spectrum of biopolymer powder without methionine is shown in Figure 3a. In this spectrum, we see a broad band with a maximum absorption peak around 300 nm. This region mainly reflects the presence of organic species, which we can attribute to the formation of different polysaccharides in the sample. Also, Figure 3a shows the spectrum of the biopolymer powder with methionine, where we observe three characteristic bands for methionine at wavelengths below 400 nm. Additionally, there is a peak at 455 nm, which indicates the oxidized form of methionine [24]. This suggests that part of the methionine bound to the polymer has undergone oxidation due to the neutralization process used.

On the other hand, the absorption spectra of powder samples in suspension (0.04 g/mL of the biopolymer in water) were obtained with a UV-VIS GENESYS 180, Thermo Fisher Scientific Inc., Waltham, MA, USA, too. Figure 3b shows the spectrum of the biopolymer without methionine in suspension (black line); in this graph, a rough line over the UV-VIS-NIR range from 330 nm to 800 nm can be observed. This region mainly reflects the presence of organic species, which we can attribute to the formation of different polysaccharides in the sample. In the same panel, the (red line) shows the spectrum of the biopolymer with methionine in suspension, where a smooth line over the visible and near-infrared region can be observed. This suggests that part of the methionine bound to the polymer underwent oxidation due to the neutralization process used [24]. As shown in this figure, the sample without methionine was composed of various elements and was not homogeneous, while the sample that had methionine formed a homogeneous and more stable compound. When we compared both samples, in powder and in suspension, we observed a diminution of the absorbance in the suspension samples due to less concentration of methionine.

### 3.2. Photoluminescence

**Figure 4:**
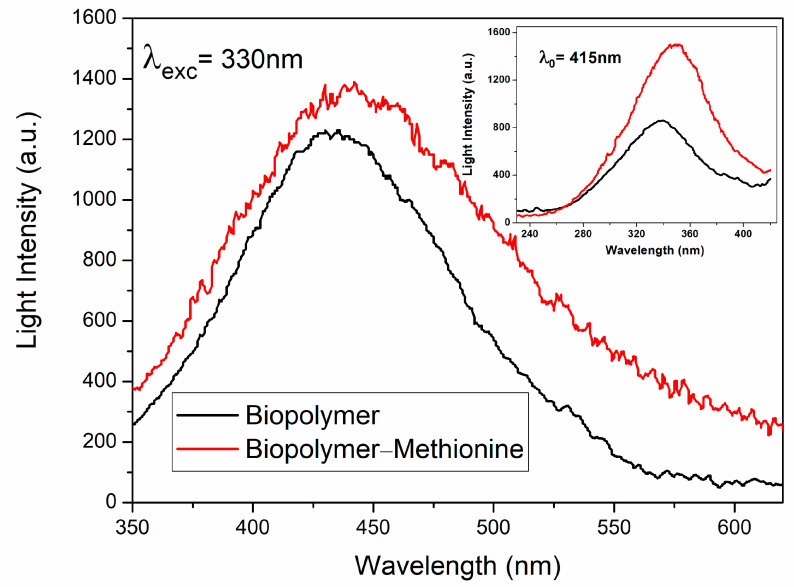
Emission spectra of diluted powdered biopolymer natural with methionine (red line) and without methionine (black line), both measured at λ_exc_ = 330 nm. The excitation spectra at λ_0_ = 415 nm of both samples are shown in the inset, too.

Fluorescence spectra were measured with a spectrophotometer (Fluorescence), PerkinElmer Inc., 650-10S Shelton, CT, USA at room temperature, in the range from 350 nm to 620 nm. To obtain a clear emission measurement, the biopolymer powder was diluted in double-distilled water; after that, the biopolymer in solution was placed in a quartz cuvette to measure the photoluminescence. Each characteristic emission spectrum of the biopolymer was recorded following excitation at a wavelength of 330 nm (Figure 4). The one with methionine (red line) and the one without methionine (black line) consist of broad bands from 350 nm to 600 nm with maxima intensities at 437 nm and 424 nm, respectively. It can be observed that the sample with methionine resulted in a wider band, which was expected because of the added element and the presence of an amorphous material.

The excitation spectrum of the banana peel biopolymer with methionine can be observed to be better defined and relatively more intense. The inset of Figure 4 shows the excitation spectra of the biopolymers with (red line) and without methionine (black line), peaking at approximately 348 nm and 338 nm, respectively, too.

### 3.3. FTIR Spectra

**Figure 5:**
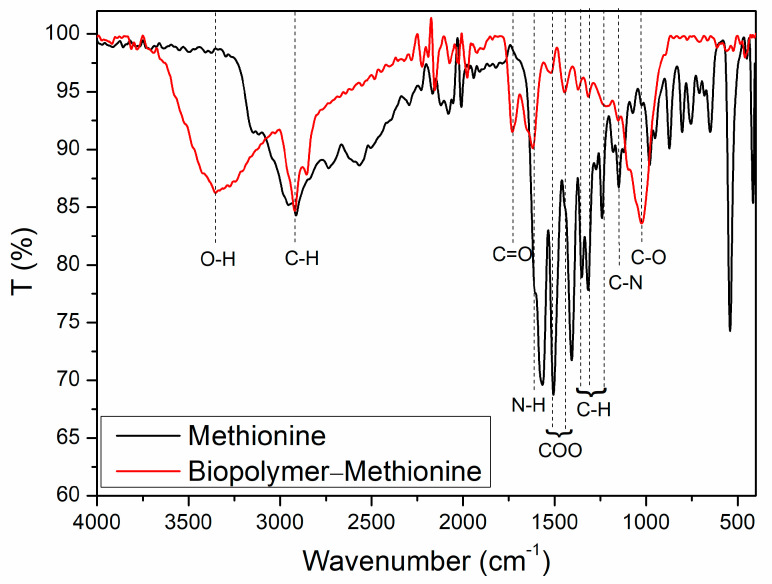
FTIR spectra: methionine (black line) and the biopolymer–methionine product (red line).

We obtained the infrared spectrum of the biopolymer with methionine and compared it with the spectrum of the methionine used in the synthesis. This comparison allowed for the identification of overlapping methionine bands within the polymer banana peels (Figure 5).

In the spectrum of the biopolymer, we observed a broad band at around 3300 cm^−1^, typical of O-H stretching vibrations. The first noticeable overlap occurs at approximately 2920 cm^−1^, corresponding to the symmetric stretching vibrations of C-H bonds. These vibrations are characteristic of the CH_2_ or CH_3_ groups present in the molecules.

We observed a band at 1730 cm^−1^, which may be attributed to the symmetric stretching of carbonyl groups. This suggests the formation of ester bonds with the amino acid, a finding supported by the symmetric and asymmetric stretching bands of carboxylates found in ester groups. Additionally, there is a band at 1150 cm^−1^, related to the symmetric stretching vibrations of primary amines, which also appear in the methionine spectrum [25].

Finally, we observed an important band at 1070 cm^−1^, indicating the vibration of carbon–oxygen–carbon bonds, which structurally form bridges between monomers and indicate polymerization.

### 3.4. Thermal Analysis: TG and DTG Curves

**Figure 6:**
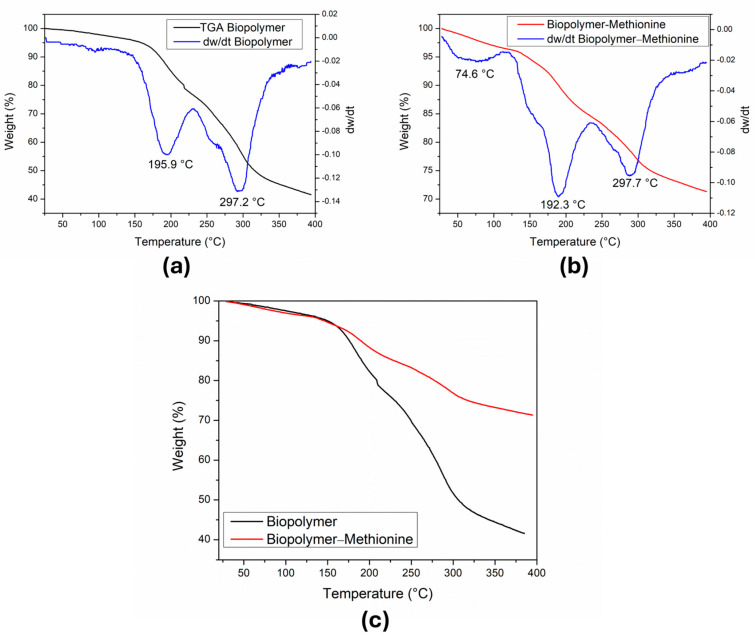
TG and DTG curves of the samples were measured from 50 °C to 450 °C. (**a**) Curve of the mass loss in the biopolymer without methionine. (**b**) Curve of the mass loss in the biopolymer with methionine. (**c**) Comparison of the mass loss in both samples.

We conducted a thermogravimetric analysis of the samples shown in Figure 6. In Figure 6a, the TG graph of the biopolymer reveals a continuous mass loss throughout the heating process and DTG shows two thermal events occurring at 195.9 °C and 297.2 °C. The initial mass (*m_i_*) used was 3.17 mg. Between room temperature ~25 and 144 °C, we observed a 4%w loss, equivalent to 0.127 mg of the *m_i_*, which corresponds to the removal of adsorbed water molecules. The sample is stable up to about 150 °C and then loses about 20%w equivalent to 0.634 mg. In the range of 150 to 232 °C (first decomposition peak), this mass loss can be attributed to the formation of water molecules from the terminal OH groups of the glucose molecules [26]. In the range from 232 to 388 °C, the sample continues to decompose, as shown by the second processing peak (297.2 °C). There is a greater loss of mass representing 34%w, which corresponds to 1.0778 mg. The total mass loss of the biopolymer was 1.84 mg at the final temperature (394 °C) and the mass of the residue was 1.32 mg, observing a difference of 0.01 mg with respect to the *m_i_*, giving a percentage error of 0.76%.

Figure 6b shows the TG and DTG graph of the biopolymer containing methionine. The DTG also shows two peaks of the sample revision at 192.3 °C and 297.7 °C. The *m_i_* of the sample used was 7.59 mg and in the temperature range from 28 to 134 °C, there was a loss of 4%w, which corresponds to 0.304 mg of the *m_i_*, and was attributed to the loss of water molecules adsorbed in the sample. Between 134 and 236 °C, 11.8%w (0.896 mg) was lost and corresponds to the formation of H_2_O molecules from the terminal OH groups of the glucose molecules. In the interval from 236 to 380 °C, 12.4%w (0.941 mg) was lost due to the formation of CO_2_ from the decomposition of glucose and methionine molecules. The total mass loss in this process was 2.14 mg, and a residue of 5.42 mg was obtained. If the total mass loss is subtracted from the *m_i_*, the result is 5.45 mg, which differs from the value of the residue mass (5.42 mg) by 0.03 mg, corresponding to a 0.55% error.

Figure 6c depicts the thermal behavior of both samples and indicates the relative weight loss percentage of the pure biopolymer and the biopolymer with methionine. This difference seems to indicate that the pure biopolymer loses mass more rapidly than the biopolymer with methionine.

### 3.5. Scanning Electron Microscopy (SEM)

**Figure 7:**
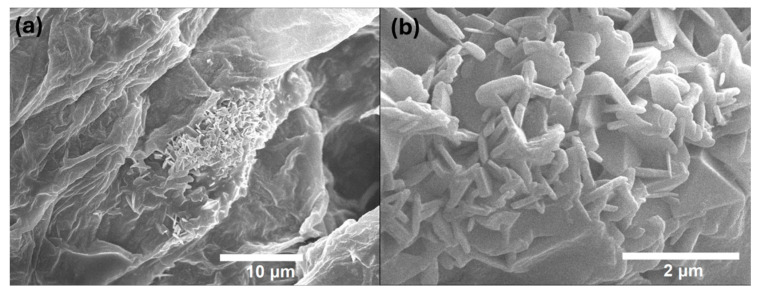
SEM images of the biopolymer. (**a**) Microscopy of the biopolymer without methionine. (**b**) Close-up of a structural cluster of the sample without methionine.

**Figure 8:**
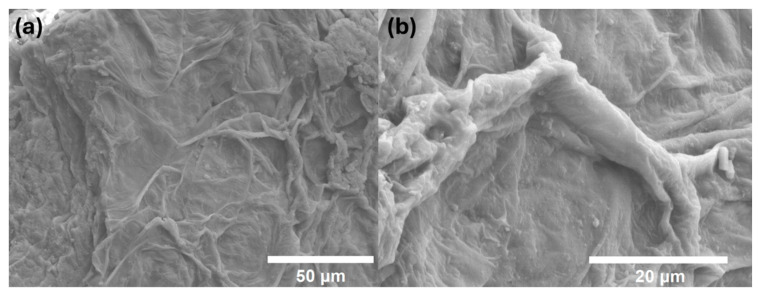
SEM images of the biopolymer with methionine. (**a**) Microscopy of the sample with methionine. (**b**) Close-up of a fiber present in the sample with methionine.

We characterized the natural biopolymer (Figure 7) and with methionine (Figure 8) obtained through scanning electron microscopy (SEM) using the JEOL Ltd., JSM-7600 F, Akishima, Japan. The images were obtained using a 15 kV configuration. We used the same equipment to characterize the energy-dispersive spectroscopy (EDS). This allowed us to obtain the final composition of the sample (Figure S1) and identify the amount of methionine in the biopolymer (Figure S2). Furthermore, minerals are preserved after the procedure such as calcium and potassium in the natural biopolymer (Figure S3).

Following this method, we carried out a physical–chemical study to characterize a biopolymer made from banana peel, with the goal of later exploring its potential for applications in the agricultural production of potatoes and in tilapia feed.

Compostable banana peel biopolymers are gaining attention as sustainable alternatives to conventional plastics. These biopolymers decompose naturally, offering environmental benefits and potential applications in various fields, including agriculture. Banana peels are rich in essential nutrients like potassium, phosphorus, and calcium, making them an excellent natural fertilizer. The bioactive compounds in the peels have shown effectiveness against various plant pathogens, reducing the incidence of diseases and promoting healthier crop growth [27]. This natural method of pathogen control can reduce the reliance on chemical pesticides, contributing to more sustainable agricultural practices.

On the other hand, it is well known that various plant-based protein sources, such as grains and vegetables, are included and tested in formulated tilapia feeds. However, it is important to note that these plant ingredients are deficient in certain essential amino acids. Wolf et al. [28] mention that methionine is a primary limiting amino acid that must be supplemented in tilapia diets to improve its growth.

## 4. Conclusions

In the chemical process to prepare the biopolymer, non-polluting reagents for humans, animals, and the environment were used, maintaining the idea of producing compounds biocompatible with nature. The acid used for hydrolyzing the banana peel was an organic acid present in several edible fruits.

The biopolymer obtained by our working group exhibits promising characteristics for its technological use in several areas of science. Currently, it is intended to be used as a carrier for nutrients such as methionine in aquaculture applications. Methionine is an essential and pivotal amino acid heavily used in animal feed, to improve health status and growth performance [29]. The inclusion of this amino acid in natural clays as a more sustainable alternative for heavy loading and efficient delivery of this nutrient has been demonstrated. In the present work, the incorporation of methionine in a biopolymer prepared from banana peel residues is reported as another innovative and environmentally friendly approach for feed applications. This will allow the nutrient to remain stable and easily available for use.

Banana peel has been detected as a sustainable feedstock for the extraction or synthesis of biologically active compounds, biopolymers, and nanomaterials with a plethora of applications in the agri-sector in pest/disease prevention, fertilizers, agrochemicals, bio-stimulants, and nutrient-delivery applications [7,30].

The biopolymer can be obtained in either powder or film form. In this study, it was prepared in powder form for application in feed in tilapia farms and in potato agricultural production. The results obtained open up significant opportunities to prepare various sustainable products from food waste and thus mitigate contamination caused by organic waste, which produces pollutants, such as methane. In addition, this approach contributes to global sustainable development.

## Data Availability

The data are contained within the article. Further queries can be directed to the corresponding authors.

## Supplementary Materials

The following supporting information can be downloaded at: https://www.mdpi.com/article/10.3390/polym17091230/s1, Figure S1: The EDS study of the biopolymer has revealed carbon and oxygen on its surface; Figure S2: The EDS study of the biopolymer with methionine has revealed carbon, oxygen and sulfur on its surface; Figure S3: The EDS study of biopolymer has revealed potassium and calcium on its surface too.
