# Supplementary material for: Banana Peel Based Cellulose Material for Agriculture and Aquiculture: Toward Circular Economy
Source: Polymers (Basel). 2025 Apr 30;17(9):1230. doi: 10.3390/polym17091230 (PMC12073761; doi:10.3390/polym17091230)
Supplement: Supplementary file 1 [file polymers-17-01230-s001.zip › polymers-3531987-supplementary.pdf]

polymers

MDPI

Supplementary information

Banana Peel Based Cellulose Material for Agriculture and Aquiculture: Toward Circular Economy

Iris N. Serratos [1], Juan Antonio García Torres [2], Jorge Luis Mendoza Téllez [1], David Silva Roy [2], Ana María Soto Estrada [1]*, Norma Elena Leyva López [3], Hervey Rodríguez González [3], Sylvie Le Borgne [4], Karla Lorena Sánchez Sánchez [5] and Rebeca Sosa Fonseca [2]*

[1] Departamento de Química, Universidad Autónoma Metropolitana-Iztapalapa, Av. San Rafael Atlixco 186, Col. Vicentina, Ciudad de México 09340, México; insa@xanum.uam.mx (I.N.S.); cbi2173048089@izt.uam.mx (J.L.M.T.)
[2] Departamento de Física, Universidad Autónoma Metropolitana-Iztapalapa, Av. San Rafael Atlixco 186, Col. Vicentina, Ciudad de México 09340, México; antonio_gt@xanum.uam.mx (J.A.G.T.); droy@xanum.uam.mx (D.S.R.)
[3] Instituto Politécnico Nacional, Centro Interdisciplinario de Investigación para el Desarrollo Integral Regional Unidad Sinaloa, Departamento de Biotecnología Agrícola, Guasave 81101, México; neleyval@ipn.mx (N.E.L.L.); hrodriguezg@ipn.mx (H.R.G.)
[4] Departamento de Procesos y Tecnología, Universidad Autónoma Metropolitana-Cuajimalpa, Vasco de Quiroga 4871, Ciudad de México 05348, México; sylvielb@cua.uam.mx
[5] Departamento de Sistemas de Información y Comunicaciones, Universidad Autónoma Metropolitana-Lerma, Av. de las Garzas 10, Lerma de Villada 52005, México; k.sanchez@correo.ler.uam.mx
* Correspondence: amse@xanum.uam.mx (A.M.S.E.); rebe@xanum.uam.mx (R.S.F.)

Keywords: compostable biopolymer; banana peels; agriculture; aquiculture; circular economy

1. EDS Study of samples

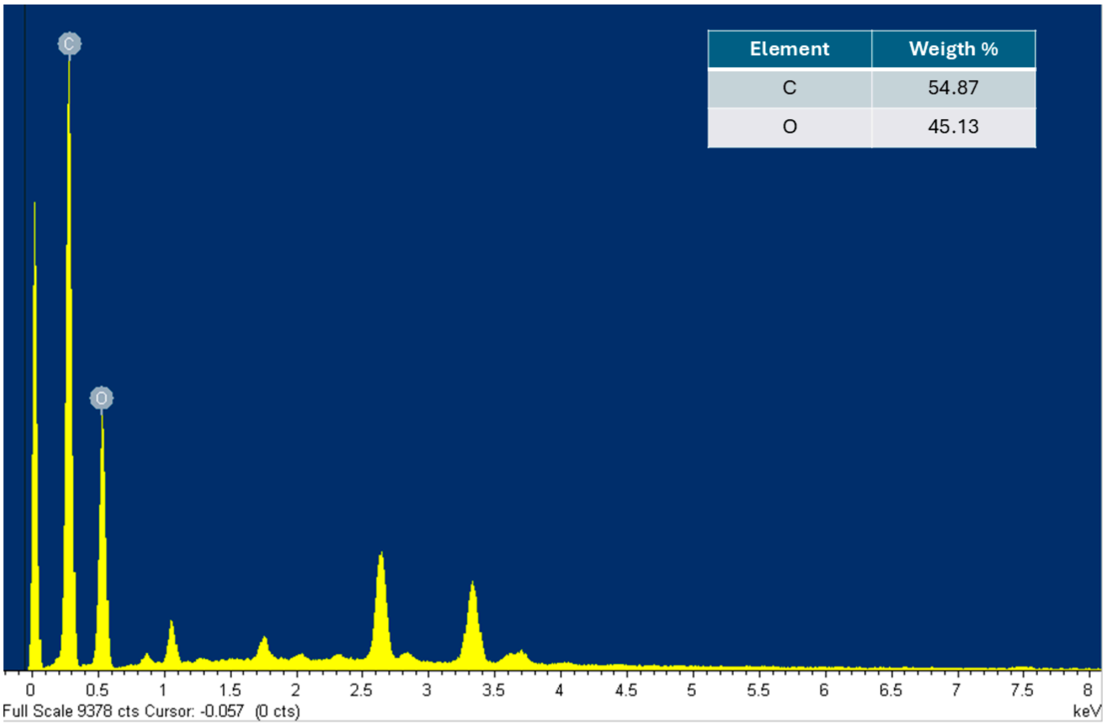

Figure S1. The EDS study of the biopolymer has revealed carbon and oxygen on its surface.

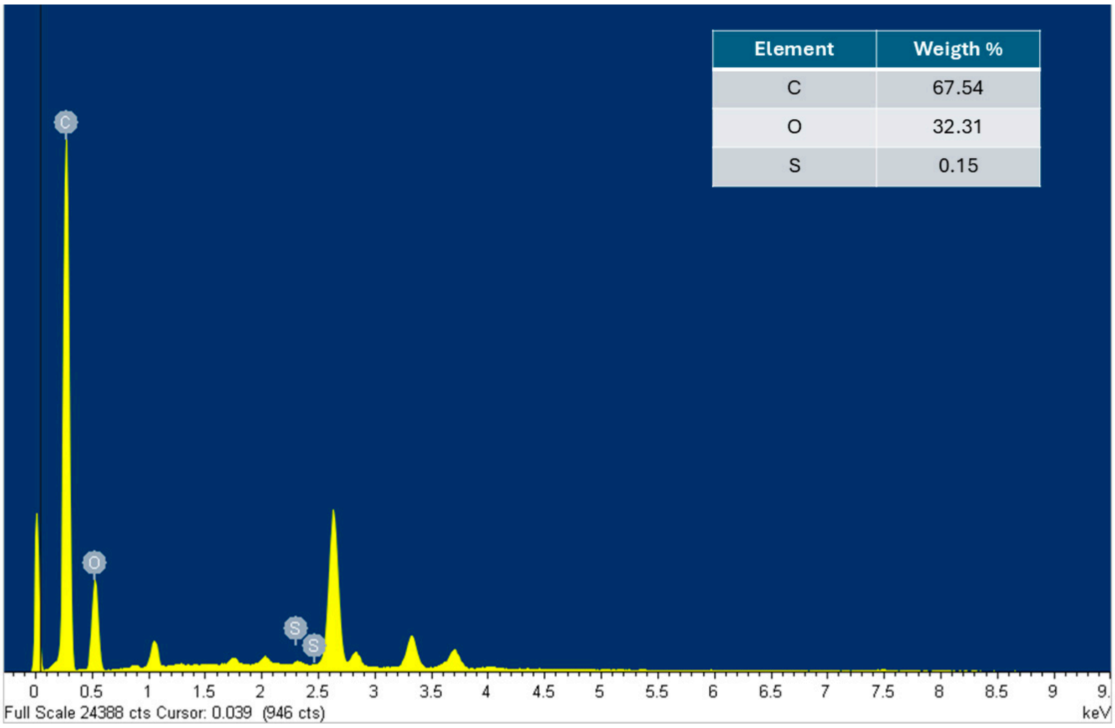

Figure S2. The EDS study of the biopolymer with methionine has revealed carbon, oxygen and sulfur on its surface.

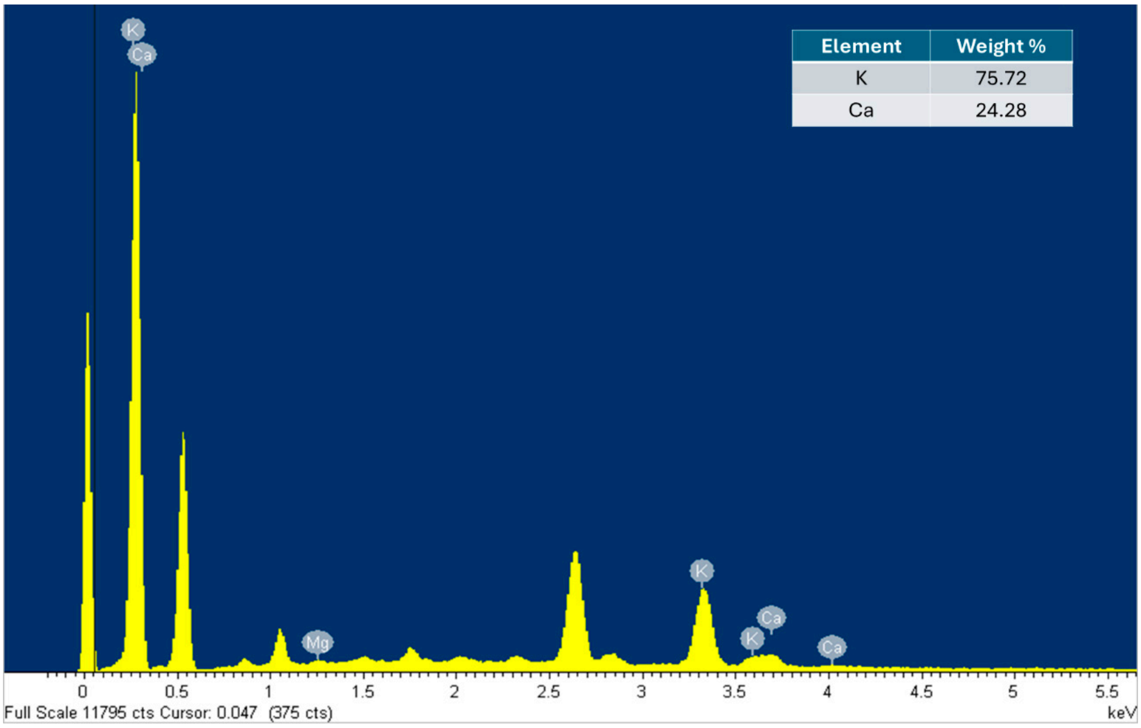

Figure S3. The EDS study of the biopolymer has revealed potassium and calcium on its surface too.

Disclaimer/Publisher's Note: The statements, opinions and data contained in all publications are solely those of the individual author(s) and contributor(s) and not of MDPI and/or the editor(s). MDPI and/or the editor(s) disclaim responsibility for any injury to people or property resulting from any ideas, methods, instructions or products referred to in the content.
